# Hip Torque Is a Mechanistic Link Between Sprint Acceleration and Maximum Velocity Performance: A Theoretical Perspective

**DOI:** 10.3389/fspor.2022.945688

**Published:** 2022-07-12

**Authors:** Kenneth P. Clark, Laurence J. Ryan

**Affiliations:** ^1^Human Performance Laboratory, Department of Kinesiology, West Chester University, West Chester, PA, United States; ^2^Independent Researcher, Dallas, TX, United States

**Keywords:** bipedal gait, sprinting, force, acceleration, running biomechanics

## Abstract

Sprinting performance is critical for a variety of sports and competitive activities. Prior research has demonstrated correlations between the limits of initial acceleration and maximum velocity for athletes of different sprinting abilities. Our perspective is that hip torque is a mechanistic link between these performance limits. A theoretical framework is presented here that provides estimates of sprint acceleration capability based on thigh angular acceleration and hip torque during the swing phase while running at maximum velocity. Performance limits were calculated using basic anthropometric values (body mass and leg length) and maximum velocity kinematic values (contact time, thigh range of motion, and stride frequency) from previously published sprint data. The proposed framework provides a mechanistic link between maximum acceleration and maximum velocity, and also explains why time constant values (τ, ratio of the velocity limit to acceleration limit) for sprint performance curves are generally close to one-second even for athletes with vastly different sprinting abilities. This perspective suggests that specific training protocols targeted to improve thigh angular acceleration and hip torque capability will benefit both acceleration and maximum velocity phases of a sprint.

## Introduction

Linear speed is a key variable determining athletic performance. Sprinting ability may differentiate athletes of various sports, positions, and playing levels (Cometti et al., 2001; Sierer et al., 2008; Vescovi, 2012; Cross et al., 2015; Wild et al., 2018; Watkins et al., 2021). The importance of linear speed has generated numerous studies examining the different phases of a sprint, as indicated by recent advances in force-velocity profiling. While prior research has demonstrated correlations between initial acceleration and maximum velocity performance (Vescovi and Mcguigan, 2008; Mendez-Villanueva et al., 2011; Buchheit et al., 2014; Clark et al., 2019), formal mechanistic connections linking the acceleration and maximum velocity phases have not been fully established.

**Figure 1:**
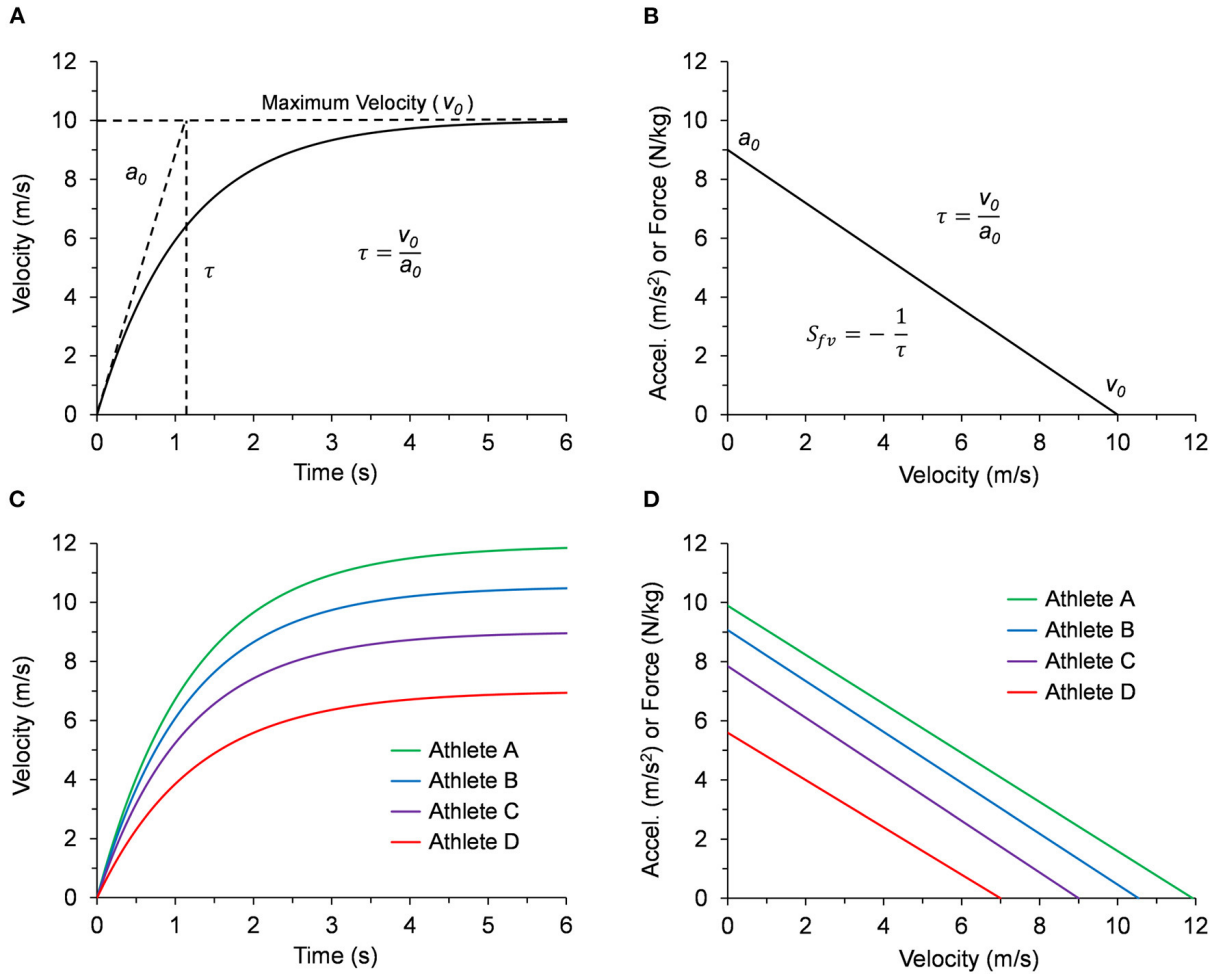
**(A)** Example graph of the exponential velocity vs. time curve during a maximum effort sprint. The slope at *t* = 0 is defined by the limit *a*_0_ and the velocity curve approaches the limit *v*_0_ according to the time constant τ = *v*_0_/*a*_0_. **(B)** The corresponding linear curve for acceleration (m/s^2^) vs. velocity, or equivalently, force relative to body mass (N/kg) vs. velocity. The negative slope of the linear acceleration vs. velocity curve *S*_*fv*_ can be defined in terms of the time constant (*S*_*fv*_ = -*a*_0_/*v*_0_ = −1/τ). **(C)** Velocity vs. time curves for all four representative athletes listed in Table 1. **(D)** Acceleration or force vs. velocity curves for all four representative athletes listed in Table 1.

As shown in Figure 1A, a runner performing a maximum effort sprint from a stationary start to maximum velocity consistently produces an exponential velocity-time curve (Furusawa et al., 1927; Di Prampero et al., 2005; Samozino et al., 2016; Cross et al., 2017; Morin et al., 2019). This curve is defined by the maximum limits of acceleration (*a*_0_) and velocity (*v*_0_) with an exponential time constant (τ = *v*_0_/*a*_0_) (Chelly and Denis, 2001). During the sprint, the average horizontal force for each step decreases linearly as running velocity increases (Samozino et al., 2016; Morin et al., 2019). As shown in Figure 1B, since maximum horizontal acceleration (m/s^2^) is equivalent to the maximum horizontal force relative to body mass (N/kg), the resultant acceleration-velocity curve can be defined by the limits *a*_0_ and *v*_0_ with a negative linear slope (*S*_*fv*_ = -*a*_0_/*v*_0_ = −1/τ). Mean values for τ and *S*_*fv*_, either directly reported or calculated from published maximum velocity and maximum force data, consistently have a magnitude of around one (τ ≈ 1 s or *S*_*fv*_ ≈−1 s^−1^) even for athletes from a variety of sports with vastly different sprinting abilities (Cross et al., 2015; Rabita et al., 2015; Slawinski et al., 2017a; Jiménez-Reyes et al., 2018; Haugen et al., 2019; Healy et al., 2019; Morin et al., 2019; Watkins et al., 2021; Edwards et al., 2022). This indicates that the acceleration and velocity performance limits are related and generally proportional.

One commonality between these distinct performance limits is that the hip requires torque capability for maximum propulsion during ground-contact of initial acceleration and also for rapidly repositioning the legs during the swing phase as maximum velocity is attained (Holmlund and von Hertzen, 1997; Nagahara et al., 2017, 2020). During a maximum effort sprint from a stationary start, horizontal force accelerates the body forward (Rabita et al., 2015) as the center of mass (COM) rises slightly (Nagahara et al., 2014). During the initial steps, the COM has angular acceleration as it rotates about the fixed contact point on the ground established by the leg, similar to an inverted pendulum (Jacobs and van Ingen Schenau, 1992). After the first few steps, runners quickly establish a thigh angular range of motion and stride frequency that is nearly constant (Nagahara et al., 2014, 2017). Runners with faster maximum sprinting speed generally exhibit larger values of maximum thigh range of motion and frequency (Clark et al., 2020). This is indicative of the rapid thigh reversal occurring at peak flexion and extension (Mann et al., 1986; Nagahara et al., 2017; Clark et al., 2020; Kakehata et al., 2021), and can be quantified by the maximum angular acceleration value (Clark et al., 2021) and corresponding torque.

The purpose of this perspective is to provide a theoretical framework that considers the torque capability of the hip as a mechanistic link between maximum acceleration and maximum velocity performance. The analysis will show that the maximum horizontal acceleration limit during ground-contact can be estimated using the maximum angular acceleration of the leg during the swing phase. The analysis will also demonstrate that the time constant τ can be expressed in terms of basic kinematic variables and maintains a mean value of around one-second over the normal range of gait values. This perspective suggests that the *a*_0_ and *v*_0_ performance limit values are fundamentally proportional and that specific training protocols targeted to improve hip torque capability would benefit both the acceleration phase and maximum velocity phase for an athlete.

## Theoretical Framework

Newton's second law for a rotating system defines the torque as the product of the moment of inertia (*I*) and angular acceleration (α) about the axis of rotation. We apply Newton's second law for two specific conditions of the sprint. The first condition is during the ground-contact phase of the first few steps of initial acceleration. Using an inverted pendulum model for general bipedal locomotion, torque at the hip joint results in a rotation of the line from the fixed contact point on the ground to the body's COM (Raibert et al., 1989; McGeer, 1990; Pratt, 2000). Thus, during acceleration, the body's COM has forward translation and angular acceleration as it sweeps through an angle relative to the ground-contact point of the leg (Jacobs and van Ingen Schenau, 1992). The second condition is during the swing phase at maximum velocity. Using a sinusoidal model for thigh motion, the angular acceleration at the hip joint approaches maximum values as the thigh reaches peak flexion and extension (Clark et al., 2021). Our perspective is that the torque limit at the hip joint is equivalent for the first and second conditions described above:


(1) $$\begin{array}{r} I_{com}\;\alpha_{com}=I_{leg}\;\alpha_{leg} \end{array}$$

where *I*_*com*_ is the moment of inertia of the body COM (kg●m^2^), α_*com*_ is the maximum angular acceleration of the COM (rad/s^2^), *I*_*leg*_ is the moment of inertia of the extended leg (kg●m^2^), and α_*leg*_ is the maximum angular acceleration of the leg (rad/s^2^).

The linear acceleration of the COM when the leg is in contact with the ground is:


(2) $$\begin{array}{r} a_{0}=\alpha_{com}\;L_{leg}=\;\frac{I_{leg}\;\alpha_{leg}}{I_{com}}\;L_{leg} \end{array}$$

where *a*_0_ is the maximum propulsive acceleration limit (m/s^2^), α_*com*_ is the maximum angular acceleration of the COM derived from Equation 1, and *L*_*leg*_ is leg length (m).

The moment of inertia of the body as represented by the point COM is:


(3) $$\begin{array}{r} I_{com}=\;m_{b}\;L_{leg}^{2} \end{array}$$

where *m*_*b*_ is body mass (kg).

While running at or near maximum velocity, each thigh rotates about the hip axis with sinusoidal motion (Mann et al., 1986; Clark et al., 2020). The angular motion of the thigh coincides with the angular motion of the leg when extended during swing phase. Thus, the maximum angular acceleration of the leg at peak flexion or extension is (Clark et al., 2021):


(4) $$\begin{array}{r} \alpha_{leg}=\alpha_{\max}=2\;\pi^{2}\;\theta_{t}\;f_{str}^{2} \end{array}$$

where α_*max*_ is the maximum angular acceleration of the thigh (rad/s^2^), θ_*t*_ is the total thigh range of motion (rad) and *f*_*str*_ is the stride frequency which is one-half the step frequency (strides/s or Hz).

The moment of inertia for the extended leg is:


(5) $$\begin{array}{r} I_{leg}=m_{leg}\;\rho^{2}\;=0.161\;m_{b}\;{\left(0.560\;L_{leg}\right)}^{2}\approx 0.05\;m_{b}\;L_{leg}^{2} \end{array}$$

where *m*_*leg*_ is leg mass (kg) and ρ is the radius of gyration from the proximal end (m). Standard anthropometric data are used for ρ = 0.560●*L*_*leg*_ and *m*_*leg*_ = 0.161●*m*_*b*_ (Winter, 2009, Table 4.1 for the total leg).

The relationships from Equations 3–5 can then be inserted into Equation 2 for the maximum propulsive acceleration limit:


(6) $$\begin{array}{r} a_{0}=\;\frac{\left(0.05\;m_{b}\;L_{leg}^{2}\right)\left(2\;\pi^{2}\;\theta_{t}\;f_{str}^{2}\right)}{\left(m_{b}\;L_{leg}^{2}\right)}\;L_{leg}\;\approx \;L_{leg}\;\theta_{t}\;f_{str}^{2} \end{array}$$

Thus, *a*_0_ can be calculated from leg length and maximum velocity kinematic values of thigh range of motion and stride frequency. The resultant value for *a*_0_ can also be equivalently expressed in units of normalized force (*F*_0_/*m*_*b*_, N/kg) using Newton's second law.

Velocity can be defined in terms of basic gait variables using the relationship (Cavagna et al., 1976):


(7) $$\begin{array}{r} v_{0}=\frac{L_{c}}{t_{c}}\;\approx \;\frac{L_{leg}}{t_{c}} \end{array}$$

where *v*_0_ is the maximum velocity limit (m/s), *L*_*c*_ is the ground-contact length (m), *t*_*c*_ is the ground-contact time (s), and *L*_*c*_ ≈ *L*_*leg*_.

The time constant (τ) for the linear acceleration-velocity curve and the exponential velocity-time curve (Figure 1) can be expressed as:


(8) $$\begin{array}{l} \tau =\;\frac{v_{0}}{a_{0}}\;=\;\frac{v_{0}}{L_{leg}\;\theta_{t}\;f_{str}^{2}}\;=\;\frac{\;L_{c}}{\;t_{c}\;L_{leg}\;\theta_{t}\;f_{str}^{2}}\\ \;=\;\frac{\;L_{c}/L_{leg}}{\;t_{c}\;\theta_{t}\;f_{str}^{2}}\;\approx \;\frac{1}{\;t_{c}\;\theta_{t}\;f_{str}^{2}} \end{array}$$

using Equations 6-7. Thus, τ can be calculated from three basic kinematic gait variables (*t*_*c*_, θ_*t*_, and *f*_*str*_) which are measured during the maximum velocity phase of the sprint.

## Performance Calculations Using the Theoretical Framework

In the first section of Table 1, we evaluated Equation 8 across an array of input values for *t*_*c*_, θ_*t*_, and *f*_*str*_ based on previously published data (Nagahara et al., 2014; Mann and Murphy, 2018; Murphy et al., 2021; Clark et al., 2020) to establish the general range of τ values under normal sprint conditions. Note that mathematically, stride frequency is a function of contact and flight time, and given relatively consistent flight times across a range of top speeds (Weyand et al., 2000), briefer *t*_*c*_ will be associated with faster *f*_*str*_. The listed values for θ_*t*_ represent a maximum thigh range of motion ranging from 1.5 to 1.9 radians (approximately 85 to 110 degrees). Across the array of input values, the resultant τ values maintained a relatively narrow range from 0.94 to 1.29 s with a mean value of 1.11 s.

**Table 1:** Performance calculations using the theoretical framework.

**Evaluation of Equation 8 across a range of fundamental gait parameters**									
	**Input variables**						**Output variables**		
	***t**_***c***_* **(s)**		***θ_*t*_*** **(rad)**		***f**_***str***_* **(Hz)**		***τ*** **(s)**		
	0.090		1.50		2.40		1.29		
	0.090		1.70		2.40		1.13		
	0.090		1.90		2.40		1.02		
	0.110		1.50		2.20		1.25		
	0.110		1.70		2.20		1.10		
	0.110		1.90		2.20		0.99		
	0.140		1.50		2.00		1.19		
	0.140		1.70		2.00		1.05		
	0.140		1.90		2.00		0.94		
	**Calculated performance outputs for four athletes using representative data**								
**Input variables**							**Output variables**		
**Athlete**	***h**_***b***_* **(m)**	***m**_***b***_* **(kg)**	***L**_***leg***_* **(m)**	***t**_***c***_* **(s)**	***θ_*t*_*** **(rad)**	***f**_***str***_* **(Hz)**	***v**_**0**_* **(m/s)**	***a**_**0**_* **(m/s**^**2**^**)**	***τ*** **(s)**
A	1.80	75.0	0.95	0.080	1.8	2.4	11.9	9.9	1.21
B	1.65	63.0	0.87	0.083	1.8	2.4	10.5	9.1	1.16
C	1.80	75.0	0.95	0.106	1.7	2.2	9.0	7.8	1.15
D	1.65	63.0	0.87	0.125	1.6	2.0	7.0	5.6	1.25

*Variables: h_b_ = body height, m_b_ = body mass, L_leg_ = leg length, t_c_ = ground-contact time at maximum velocity, θ_t_ = total thigh range of motion at maximum velocity, f_str_ = stride frequency at maximum velocity, v_0_ = maximum velocity limit, a_0_ = maximum sprint acceleration limit equivalent to F_0_ normalized to body mass (N/kg), and τ = time constant. Values for input variables t_c_, θ_t_, and f_str_ are based on Nagahara et al. (2014); Mann and Murphy (2018); Clark et al. (2020) and Murphy et al. (2021) to correspond with maximum velocities ranging from approximately 7.0 to 12.0 m/s*.

*Output variables are calculated based on input variables using Equations 6–8*.

In the second section of Table 1, we calculated outputs for *v*_0_, *a*_0_, and τ using inputs *h*_*b*_, *m*_*b*_, *L*_*leg*_, *t*_*c*_, θ_*t*_, and *f*_*str*_ for four athletes with representative data. Anthropometric data for height (*h*_*b*_) and body mass (*m*_*b*_) were based on a body mass index (BMI = *m*_*b*_*/h*${}_{b}^{2}$) of ~23 kg/m^2^ and leg length was calculated as *L*_*leg*_ = 0.53●*h*_*b*_ (Winter, 2009, Figure 4.1). Kinematic values for *t*_*c*_, θ_*t*_, and *f*_*str*_ were based on previously published data (Nagahara et al., 2014; Mann and Murphy, 2018; Murphy et al., 2021; Clark et al., 2020) for fast, intermediate, and slow athletes over a range of body dimensions. The resultant τ values maintained a range from 1.15 to 1.25 s across the range of input values. The different sprint performances from the representative athletes are illustrated by the velocity-time curves in Figure 1C and the acceleration-velocity curves in Figure 1D.

The resulting output values in Table 1 are in close agreement with previously published data. Values for *a*_0_ or normalized *F*_0_ (N/kg) are similar to those from several recent experimental investigations (Cross et al., 2015; Rabita et al., 2015; Slawinski et al., 2017a; Haugen et al., 2019; Morin et al., 2019, 2021; Watkins et al., 2021). Values for τ are slightly greater than one-second, agreeing with experimentally determined values (Healy et al., 2019; Morin et al., 2019), or values calculated from previously published maximum velocity and maximum force data (Cross et al., 2015; Rabita et al., 2015; Slawinski et al., 2017a; Haugen et al., 2019; Watkins et al., 2021; Edwards et al., 2022).

## Discussion

### Theoretical Framework Considerations

The linear force-velocity relationship during maximum effort sprinting (Figure 1B) has been studied and validated for almost a century (Furusawa et al., 1927). The initial push-off step in a sprint start utilizes both legs before transitioning to one leg to generate the propulsive force. Subsequent steps include both propulsive and braking forces as the runner moves faster while transitioning into a more upright position. The average horizontal force during each step decreases linearly until it approaches zero near maximum velocity. Mathematically, the limit *F*_0_ is the projected y-axis intercept where the initial velocity is zero (Figure 1B). Our framework estimates this limit and establishes *F*_0_ as the maximum propulsive force that can be applied by each leg. The corresponding limit of acceleration (*a*_0_ = *F*_0_/*m*_*b*_) can then be determined.

In a typical field setting, the velocity-time curve is measured using a timing system, radar or laser gun, robotic tether system, or similar technology. The limit values for *a*_0_, *v*_0_, and τ = *v*_0_/*a*_0_ are then determined using least-squares regression of the measured velocity curve (Figure 1A). Several external factors may cause measured values for *a*_0_ to differ from our estimated limit values. Lower measured values of *a*_0_ may occur if there is insufficient friction between the ground and foot, if the athlete starts from a standing two-point stance (Slawinski et al., 2017b), or when testing developmental athletes (Cahill et al., 2020; Feser et al., 2021) who may have less experience in starting technique. Additionally, athletes with higher estimated *a*_0_ capability may control the initial horizontal acceleration to optimize the projection angle of the COM (Kugler and Janshen, 2010), also resulting in lower measured values of *a*_0_. Another important factor is the initiation of the timing system. If the timing mechanism is triggered from a timing gate or when the athlete's base hand lifts off the ground, higher measured values of *a*_0_ may be obtained if force production and initial forward acceleration of the COM begin prior to the timing trigger (Clark et al., 2019).

In addition to the above considerations, there were several simplifications used in this framework that enabled fundamental gait parameters to evaluate the performance limits: (1) during the ground-contact phase in initial acceleration, the body was considered as a point COM sweeping through an angle established by the line from the fixed contact point on the ground to the body's COM; (2) during the swing phase at maximum velocity, angular acceleration was derived from the sinusoidal motion of each thigh rotating about the hip axis; (3) leg length was considered equivalent to *L*_*leg*_ during the peak propulsive period in the ground-contact phase and during the regions of maximum angular acceleration in the swing phase; (4) the distance traveled by the COM during ground-contact was considered equivalent to *L*_*leg*_; and (5) air friction was considered negligible. These approximations allowed a simple analytical framework, requiring only inputs of anthropometric values (*m*_*b*_, *L*_*leg*_) and maximum velocity kinematic values (*t*_*c*_, θ_*t*_, *f*_*str*_), to establish hip torque as a mechanistic link between maximum acceleration and maximum velocity performance.

### Practical Applications

Prior investigations have established a correlation between performance in the acceleration and maximum velocity phases, although the strength of this relationship may depend on the demographics of the sample population (Vescovi and Mcguigan, 2008; Mendez-Villanueva et al., 2011; Buchheit et al., 2014; Clark et al., 2019). While correlational analyses may be sample-dependent, our framework provides a mechanistic link between acceleration and maximum velocity phases. Table 1 demonstrates the relatively narrow range of τ values calculated from normal maximum velocity kinematic values based on previously published data. Furthermore, despite the different sprint performances expected from the representative athletes as illustrated by the velocity-time curves in Figure 1C, the acceleration-velocity curves of the representative data in Figure 1D demonstrate the general proportionality of *a*_0_ and *v*_0_ with a range of τ values slightly greater than one-second (Table 1). Therefore, this framework presents a macro-level explanation for why group mean τ and *S*_*fv*_ values are usually around one (τ ≈ 1 s or *S*_*fv*_ ≈−1 s^−1^) agreeing with experimentally determined values (Healy et al., 2019; Morin et al., 2019), or values calculated from previously published maximum velocity and maximum force data (Cross et al., 2015; Rabita et al., 2015; Slawinski et al., 2017a; Haugen et al., 2019; Watkins et al., 2021; Edwards et al., 2022).

However, inter-individual differences in force-velocity profiles certainly exist within athletic populations, and are important for specific training prescription (Morin and Samozino, 2016). Our framework could potentially supplement existing force-velocity profiling methods by determining whether athletes are performing up to their *F*_0_ or *a*_0_ capabilities. For an individual athlete, input parameters can be determined from anthropometric dimensions and kinematic measurements at maximum velocity, generating outputs of *a*_0_ to estimate limits of acceleration performance. If the athlete's measured *a*_0_ does not align with the estimated output value, it may indicate that acceleration performance can be improved through additional practice and increased motor skill competency. This may be especially true for developmental athletes who have the capacity to produce high values of α_*max*_ during maximum velocity sprinting, but may not have the technical proficiency to translate this hip torque into high levels of *F*_0_ or *a*_0_ values during the sprint start.

Finally, our analysis suggests that both the acceleration and maximum velocity phases of a sprint will benefit from specific training protocols targeted to improve hip torque capability. Classic research has indicated that extensor forces get transmitted from the hip to the ground in a proximal-to-distal sequence (Jacobs and van Ingen Schenau, 1992; Jacobs et al., 1996). More recent investigations have demonstrated that the hip extensor (hamstring) muscles play an important role in horizontal force production during sprint accelerations (Morin et al., 2015). Additionally, both the hip extensors and hip flexors are important for the rapid thigh reversal that occurs during the swing phase in maximum velocity sprinting (Dorn et al., 2012; Clark et al., 2021; Kakehata et al., 2021). Therefore, interventions aimed at enhancing an athlete's α_*max*_ via increased hip torque capability (Deane et al., 2005; Macadam et al., 2020) may be beneficial, and warrant further investigation.

## Summary

An athlete performing a maximum effort sprint from a stationary start approaches maximum velocity with an exponential velocity vs. time curve. This curve is defined by the limits of acceleration and velocity with an exponential time constant expressed as the ratio of these limits. With nearly a century of observations, the time constant is consistently around one-second even for athletes from a variety of sports with vastly different sprinting abilities. This indicates that the acceleration and velocity limits are related and generally proportional. No theory has formally defined an underlying mechanism to account for this proportionality. Our perspective is that the torque capability of the hip is a mechanistic link between the acceleration and velocity limits. Drawing on previously published models and performance data, a theoretical framework was presented here that derived a simple equation for the time constant based on fundamental gait variables. We demonstrated that the time constant maintains a mean value of around one-second over the physical range of sprinting gait values. Therefore, our perspective suggests that the limit values are fundamentally proportional and that training protocols targeted to improve hip torque capability would benefit both acceleration and velocity performance for an athlete.

## Data Availability Statement

The original contributions presented in the study are included in the article/supplementary material, further inquiries can be directed to the corresponding author.

## Author Contributions

KC and LR contributed equally to the development, drafting, writing, reviewing, and editing of this theoretical perspective. Both authors contributed to the article and approved the submitted version.

## Conflict of Interest

The authors declare that the research was conducted in the absence of any commercial or financial relationships that could be construed as a potential conflict of interest.

## Publisher's Note

All claims expressed in this article are solely those of the authors and do not necessarily represent those of their affiliated organizations, or those of the publisher, the editors and the reviewers. Any product that may be evaluated in this article, or claim that may be made by its manufacturer, is not guaranteed or endorsed by the publisher.

## Abbreviations

*a*_0_: maximum propulsive acceleration limit (m/s^2^)
BMI: body mass index (kg/m^2^)
COM: center of mass
*F*_0_: maximum propulsive force limit (N)
*f*_*str*_: stride frequency (one-half the step frequency) (strides/s or Hz)
*h*_*b*_: body height (m)
*I*_*com*_: moment of inertia of the COM (kg●m^2^)
*I*_*leg*_: moment of inertia of the extended leg (kg●m^2^)
*L*_*c*_: ground-contact length (m)
*L*_*leg*_: leg length (m)
*m*_*b*_: body mass (kg)
*m*_*leg*_: leg mass (kg)
*S*_*fv*_: slope of acceleration-velocity curve (s^−1^)
*t*: time (s)
*t*_*c*_: ground-contact time (s)
*v*_0_: maximum velocity limit (m/s)
α_*com*_: maximum angular acceleration of COM (rad/s^2^)
α_*leg*_: maximum angular acceleration of extended leg (rad/s^2^)
α_*max*_: maximum angular acceleration of thigh (rad/s^2^)
θ_*t*_: total thigh range of motion (rad)
ρ: radius of gyration from the proximal end (m)
τ: time constant of acceleration-velocity curve (s).
